# Practical Points

**Published:** 1895-09-21

**Authors:** 


### PRACTICAL POINTS.

*A Nurse's Dismissal.—There is a certain Institute in London whose rule is for nurses on resigning to give a month's notice or a month's salary. The nurses generally tender the former, which is accepted unconditionally. Usually the nurse is, at the time of giving notice, engaged on a case, which often terminates—it may be a fortnight or even longer—before her month ends. The matron then asks her if, on taking another case she will retain it till the end, no matter what the length. The nurse, in all probability, longing for her holiday, and being weary objects to taking a long case though she does not wish to leave before her time is up, and is quite willing to work until then. The matron then, without any preface, requests her to make her preparations for leaving at once, and presents her with her month's salary, thereby saving the nurse's board and lodging. She does not trouble whether that nurse has a home or friends to go to. And as, on the nurse's side, her arrangements are made dating from the end of the month, it is very often most inconvenient. Would you kindly tell me if the nurse can lay claim for her board and lodging until the termination of her agreement?—A Nurse from the above Institute.*

*** We would point out that it was decided by Mr. Justice Hill, in the case of Gordon v. Potter, 1 F. and F., page 644, that a domestic servant discharged without reason is entitled to the wages accruing up the time of her discharge, and to a calendar month's wages in addition, but not to board-wages for the month. We are not aware that the point has ever been decided, except in the case of a domestic servant, but we do not see any distinction between the case of a nurse and the case of a domestic servant which would justify a departure from the rule laid down by Mr. Justice Hill in the case above referred to.

